# Once-daily tasimelteon (VEC-162) for jet lag following transmeridian travel: A multicenter, randomized, double-blind, placebo-controlled trial

**DOI:** 10.3389/fneur.2022.901467

**Published:** 2022-09-15

**Authors:** Christos M. Polymeropoulos, Vasilios M. Polymeropoulos, Emily L. Czeisler, Michaela A. Fisher, Sandra P. Smieszek, Changfu Xiao, Gunther Birznieks, Mihael H. Polymeropoulos

**Affiliations:** Vanda Pharmaceuticals Inc., Washington, DC, United States

**Keywords:** Jet Lag Disorder, circadian rhythm, tasimelteon, sleep, circadian, HETLIOZ®, jet lag

## Abstract

Jet Lag Disorder is a Circadian Rhythm Sleep-Wake Disorder resulting from a misalignment of the endogenous circadian clock and the sleep and wake pattern required by a change in time zone. Jet lag is most severe following eastward travel. This multicenter, randomized, placebo-controlled clinical trial (JET) assessed the physiological mechanism of jet lag induced by a real-life transmeridian flight and evaluated the efficacy of tasimelteon—a circadian regulator acting as a dual melatonin receptor agonist, in the treatment of Jet Lag Disorder (JLD). Tasimelteon-treated participants slept 76 min longer on Night 3 during their second trip (evaluation phase) as compared to their first (observational phase). Over the three travel nights evaluated, transmeridian jet travelers in the tasimelteon group slept 131 min more (TST_2/3_) than those in the placebo group. The JET study demonstrated clinically meaningful improvements in nighttime sleep and daytime alertness in both objective and subjective measures as well as global functioning after a real-world flight. These results suggest that tasimelteon can be an effective therapeutic tool to treat JLD in the context of transmeridian travel.

## Introduction

The term “jet lag” was first used in a *Los Angeles Times* article on February 13, 1966 according to the Smithsonian's *Air & Space Magazine*. Horace Sutton, a magazine columnist and travel author, wrote, “If you're going to be a member of the Jet Set and fly off to Katmandu for coffee with King Mahendra, you can count on contracting Jet Lag, a debility not unakin to a hangover. Jet Lag derives from the simple fact that jets travel so fast they leave your body rhythms behind.” While jet lag has been formally defined since then, Sutton's initial characterization of jet lag speaks to the fundamental underlying mechanism of Jet Lag Disorder (JLD) being the misaligned circadian system and to the debilitating effects of this misalignment on those who travel across time zones.

Today, JLD is a globally recognized Circadian Rhythm Sleep-Wake Disorder (CRSWD), ICD-10-CM code: G47.25, defined in the *International Classification of Sleep Disorders* (Edition 3, 2014) (1) according to the following Essential Features:

Essential Features

Jet lag disorder is characterized by a temporary mismatch between the timing of the sleep and wake cycle generated by the endogenous circadian clock and that of the sleep and wake pattern required by a change in time zone. Individuals complain of disturbed sleep, sleepiness and fatigue, and impaired daytime function. The severity and duration of symptoms is dependent on the number of time zones traveled, the ability to sleep while traveling, exposure to appropriate circadian times cues in the new environment, tolerance to circadian misalignment when awake during the biological night, and the direction of the travel. Eastward travel (requiring advancing circadian rhythms and sleep-wake hours) is usually more difficult to adjust to than westward travel.

This ICSD-3 definition suggests that the symptoms of JLD are generated by circadian misalignment, the inevitable consequence of crossing time zones too rapidly for the circadian system to keep pace (1).

The intensity and duration of the disorder are related to: (1) the number of time zones crossed, (2) the direction of travel (eastward vs. westward), (3) the ability to sleep while traveling, (4) the availability and intensity of local circadian time cues (i.e., daylight), and (5) individual differences in phase tolerance. Sleep disorder, including insomnia and daytime sleepiness, is the primary complaint reported by travelers experiencing jet lag (2).

The Essential Features of JLD are related to disturbance of sleep and wakefulness due to the temporary mismatch between one's central circadian clock and that of the new time zone, which is thought to be the underlying mechanism of JLD. The misalignment between the endogenous circadian rhythm of sleep propensity and the required scheduling of sleep in the new time zone causes an inability to sleep at night in the new time zone and to remain awake during local daytime. Circadian rhythms are regulated by an internal time system. The central circadian clock—or body clock—is located in the suprachiasmatic nucleus (SCN) and is involved in regulating hormone release, body temperature, and other daily cycles (3). The rhythmic releases of melatonin produced by the pineal gland, of cortisol produced by the adrenal glands, and of the sleep-wake cycle are known to be under the regulatory control of the SCN (3). Melatonin is secreted exclusively at night in both nocturnal and diurnal animals and represents the biochemical signal of darkness.

Melatonin begins to rise ~1 h before bedtime, peaks ~4 h before awakening, and declines until ~1 h after awakening (4). Difficulties awakening and daytime sleepiness and fatigue occur because wake time also occurs at an earlier biological time when the circadian clock is still promoting sleep (i.e., during the traveler's biological night) (1). Misalignment between the body clock and the new time zone challenges our ability to sleep during the nighttime and remain awake during the daytime because the sleep promoting hormone melatonin is released when we are scheduled to be awake in the new time zone while the wake maintenance zone—a strong circadian arousal signal prior to the dim light melatonin onset—may coincide with our desired bedtime.

JLD affects approximately two-thirds of more than 100 million travelers who fly across more than two time zones, particularly those traveling in an eastward direction of travel. An even higher fraction of the 30 million Americans who cross five or more time zones annually are affected by jet lag (5).

There is no FDA-approved therapy for jet lag. The current standard of care is limited and includes methods to shift circadian rhythms prior to departure and post-arrival, as well as medications to promote sleep and/or alertness as needed (2, 5). Appropriately timed bright light exposure prior to travel has been shown to shift circadian rhythms in the desired direction, but requires high motivation and strict compliance with a prescribed light-dark schedule (2, 5). Circadian rhythms can also be shifted post-arrival and there is some evidence that melatonin administered at the appropriate time can reduce the symptoms of jet lag and improve sleep after crossing multiple time zones (6, 7). While hypnotics can be used to stimulate sleep and stimulants can be used to promote alertness, the potential negative side effects must be weighed against their ultimate usefulness.

Though JLD is a transient condition, it can nonetheless cause significant morbidity and even mortality due to accidents. Automobile accidents have been caused by JLD following transmeridien travel (8).

Tasimelteon is a circadian regulator acting as a dual melatonin receptor agonist with selective activity at the melatonin MT_1_ and MT_2_ receptors (9). Clinically, tasimelteon has been shown to regulate the timing of melatonin secretion when administered to participants in advance of the usual dim light onset of melatonin secretion. Tasimelteon was developed with the expectation that when administered at a fixed clock time, it would entrain the master body clock to a 24-h rhythm and as a result synchronize hormones like melatonin and cortisol and synchronize the sleep-wake cycle and other rhythms under control of the master clock to the 24-h day. Studies looking at tasimelteon for JLD were designed to investigate the effectiveness of tasimelteon in alleviating the symptoms of jet lag by both improving nighttime insomnia and minimizing excessive daytime sleepiness.

Tasimelteon (HETLIOZ®) has received market authorization for the treatment of Non-24-h Sleep-Wake Disorder (Non-24) (10), and for the treatment of nighttime sleep disturbances in Smith-Magenis Syndrome (SMS) (11) by the United States (US) Food and Drug Administration and also for the treatment of Non-24 in totally blind individuals by the European Medicines Agency. By acting upon the MT_1_ and MT_2_ receptors, tasimelteon may also be useful for treating JLD by exerting both a circadian regulatory effect and potentially a soporific effect by quieting the circadian drive for wakefulness at the desired sleep time.

To evaluate tasimelteon in the treatment of JLD, we conducted an observational study to characterize baseline jet lag and identify participants who experience jet lag following transmeridian travel. Study participants traveled either five or eight time zones from Washington, DC to London and San Francisco or Los Angeles to London, respectively. Transatlantic flights were chosen over a lab based phase advance procedure to most accurately study real-world jet lag. The observational baseline assessment (observational phase) was followed by a placebo-controlled interventional study (evaluation phase) focusing on the Essential Features of JLD, namely insomnia during phase-advance sleep following eastward travel, and excessive daytime sleepiness during scheduled wakefulness in the new time zone following eastward travel.

As noted in the Essential Features for JLD defined by the *International Classification of Sleep Disorders*, eastward time zone travel requires a phase advance in the timing of the scheduled sleep-wake cycle and the circadian timing system and often leads to difficulties with sleep onset as attempts to sleep are made at an earlier internal circadian time when the traveler's biological clock is promoting alertness (i.e., biological day) (ICSD-3-220-224). Jet lag is most severe following eastward travel and as such the JET study evaluated the efficacy of tasimelteon for the treatment of insomnia and excessive daytime sleepiness following eastbound transmeridian travel.

## Materials and methods

### Study design

**Figure 1:**
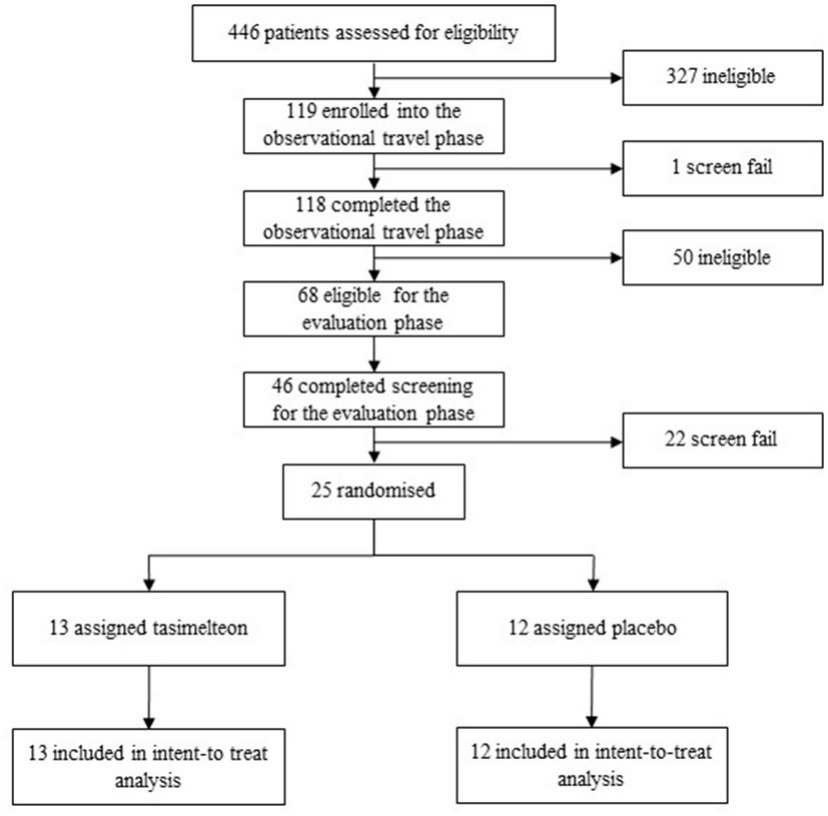
Study design.

**Figure 2:**
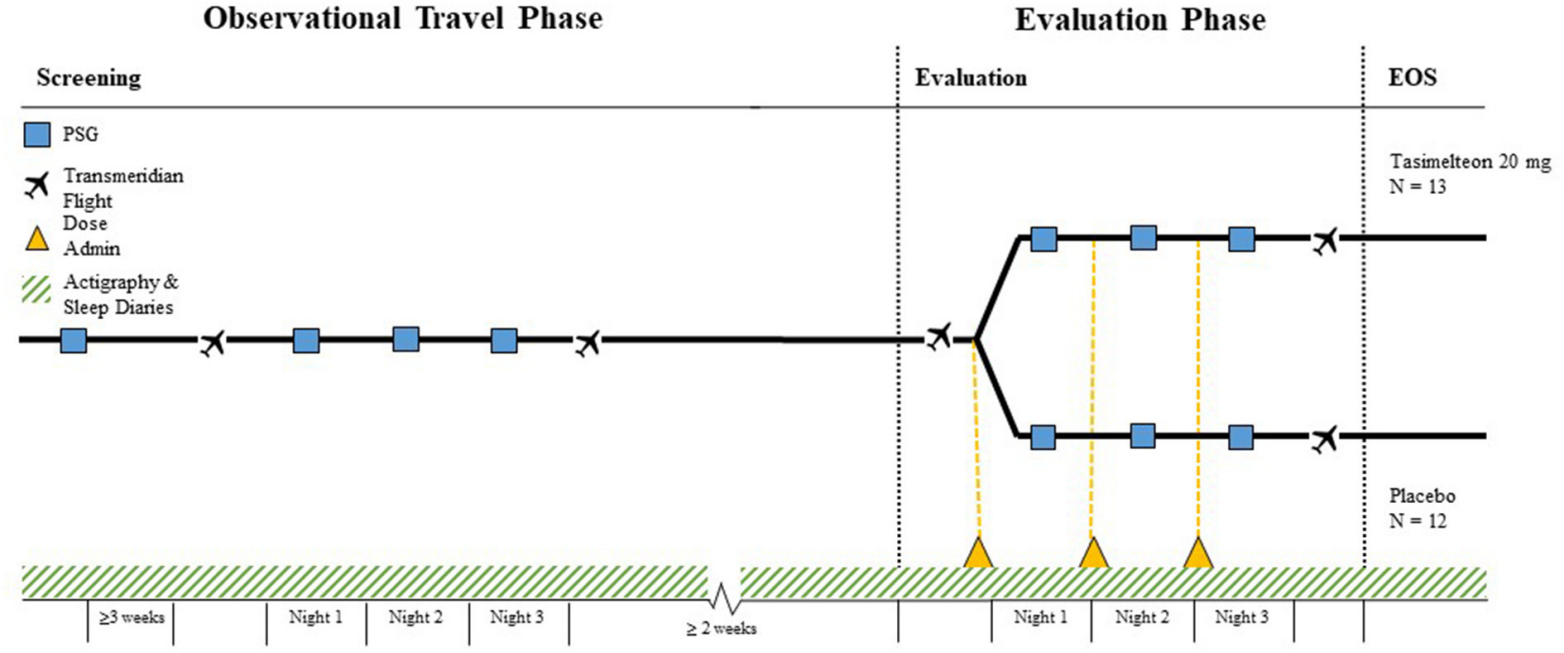
Phase advance raster plot—sample 5 h phase advance.

The JET study used a multicenter, randomized, double-blind, placebo-controlled, parallel-group design and was conducted between September 24, 2015 and November 6, 2017 at three US and three United Kingdom (UK) clinical research centers and sleep centers. The study was approved by two central Institutional Review Boards by country; the Copernicus Group IRB in the US or the Yorkshire and The Humber—Sheffield Research Ethics Committee in the UK. Screening consisted of a diagnostic polysomnography (PSG) test following a 5- or 8-h phase advance and a three-night observational travel period during which eligible participants were flown from the US to the UK on a commercial non-stop, transatlantic, over-night flight experiencing a 5- or 8-h time zone change (baseline). Evaluation consisted of a second travel period during which eligible participants were again flown from the US to the UK and were administered the study medication 30 min (±15 min) prior to bedtime, which was determined by their habitual bedtime in the originating time zone (Figures 1, 2). Participants were not scheduled to travel during the week following Daylight Savings Time (DST) in either the US or UK.

### Participants

Participants were men and women aged between 18 and 75, in good health (determined by medical and psychiatric history, physical examination, electrocardiogram, serum chemistry, and hematology) and without a history of primary insomnia or any sleep disorder, other than jet lag. Recruitment of participants was accomplished through advertisements, healthy volunteer databases, and pre-screening interviews conducted by phone script at sites in the US. Study participants provided written informed consent before any screening procedures began.

At the first study visit, participants were instructed on good sleep hygiene including keeping a regular wake time and bedtime. Participants must have had a bedtime between 21:00 and 00:00 that did not differ by more than 2 h on most nights. After the initial screening visit, a diagnostic PSG phase advance was used to rule out any exclusionary sleep disorders and to confirm whether the participant's sleep was challenged by a phase advance. The magnitude of the phase advance was timed to be equal to the planned number of time zones (five or eight) the participant would be traveling. Those whose diagnostic PSG demonstrated a sleep efficiency of ≤ 60% during the first two-thirds of the 8-h sleep opportunity (SE_2/3_) and who maintained good sleep hygiene including total nighttime sleep time between seven to 9 h on average during the previous week were eligible to continue to the observational travel period.

To be eligible for randomization in the evaluation phase the participant must have demonstrated a Jet Lag Symptom Scale (JLSS) total score of ≥4. JLSS total score was based on the sleep efficiency (score of 2 given for each night SE_2/3_ was ≤ 80%, score of 1 for each night SE_2/3_ was ≤ 85%) and Karolinska Sleepiness Scale (KSS) score (score of 2 for each day with an average KSS score of ≥6) observed during the three-night observational travel period.

### Randomization and masking

Randomization was performed using an interactive web response system (IWRS). On Night 1 of the evaluation phase, the Investigator or designee logged on to the IWRS to assign a randomization number that determined which study medication the participant was assigned. The randomization scheme was stratified by time zone within the clinical site. Participants were assigned to either 20 mg tasimelteon or placebo in a 1:1 ratio. The tasimelteon dosage form was size 1, dark blue, opaque, hard gelatin capsules with two white bars on the capsule body and cap, and filled with white to off-white powder. Placebo capsules were provided in size and appearance identical to those containing tasimelteon.

### Procedures

Subjective assessments collected *via* diary during screening included the Patient Global Impression of Severity Scale (PGI-S), the Karolinska Sleepiness Scale (KSS), and the post-sleep questionnaire (PSQ). The PGI-S was used to rate the participants' overall perceptions of the severity of their jet lag symptoms by asking participants to rate their jet lag condition at the present time on a scale of 1 “normal” to 4 “severe”. The KSS measured subjective levels of fatigue by asking participants how sleepy they felt at that moment, on a 9-point scale with 1 being “1 = extremely alert” and “9 = Very sleepy”, great effort to stay awake, fighting sleep. The PSQ was a self-reported measurement of bedtime, sleep latency, number of nighttime awakenings, wake after sleep onset (WASO), total sleep time (TST), sleep quality (SQ), number of day time naps, and the time and length of each nap.

Daily electronic diaries and wrist actigraphy watches were used to ensure compliance with eligibility parameters and to confirm habitual bedtime during the outpatient screening period.

A diagnostic PSG was conducted prior to the initial transatlantic flight. The PGI-S, KSS, and PSQ were administered after the baseline PSG. Following the initial transatlantic flight, participants underwent a three-night evaluation period in the new time zone consisting of overnight PSG and completion of the PGI-S, KSS, and PSQ assessments. No study drug was administered during this observational phase of the study.

Participants continuing to meet eligibility criteria entered the evaluation phase, which consisted of a three-night double-blind evaluation period in the new time zone, following a second transatlantic flight. Participants were put to bed at their habitual bedtime, which was determined by the median bedtime of seven nights prior to the transatlantic flight to the UK, as recorded by diary and confirmed by actigraphy. The participants' bedtime in the new time zone was ~5- or 8-h earlier than their internal-clock time in the US. Single oral dose administration of medication, tasimelteon or placebo, occurred 30 min (±15 min) prior to lights off. Tasimelteon and placebo were manufactured by Patheon Inc. in Cincinnati, Ohio, US. Participants sleep was monitored by PSG, and centrally scored in 30-s epochs.

During evaluation, assessments were taken including the PGI-S, KSS, and PSQ. Within 1 h of awakening on Day 2 and 3 of the evaluation phase, participants completed the PSQ, followed by the KSS and PGI-S 1.5 h after awakening. The KSS and PGI-S were administered six additional times throughout the day. Assessments were completed every 1.5–2.5 h after awakening. On Day 4 of the evaluation phase, participants completed the PSQ within 1 h of waking. The KSS and PGI-S were administered three times throughout Day 4 starting at 1.5 h after awakening.

### Outcomes

The prespecified primary endpoint was total sleep time in the first two-thirds (TST_2/3_) of the night(s) most likely to be disrupted as measured by PSG. Examination of the observational phase baseline data demonstrated that Night 3 was the night most disrupted with 197 min in TST_2/3_, followed by Night 1 and Night 2 with 218 and 250 min, respectively. Therefore, the primary efficacy endpoint was TST_2/3_ on Night 3 following a real-world transmeridian flight.

Secondary objective outcomes measured by PSG included TST, latency to persistent sleep (LPS), sleep efficiency (SE), WASO, total REM sleep time, total non-REM sleep time, latency to REM, and time to accumulate 30 min of REM sleep. TST was defined as the time spent sleeping between the specified time interval, such as TST_1/2_ is the TST in the first half of the night (starting from lights-out). LPS was defined as the length of time elapsed between lights-out and onset of persistent sleep. Persistent sleep was defined as the point at which 10 min of uninterrupted sleep had begun. SE was defined as the total sleep time, as a percentage of the specified time interval, such as SE_1/3_ is the sleep efficiency in the first third of the night. WASO was defined as the time spent awake between onsets of sleep and lights-on.

Secondary subjective outcomes measured by sleep diaries included TST_s_, SL_s_, SQ_s_. Secondary subjective outcomes also included the next day residual effects of tasimelteon on global functioning, as measured by PGI-S, KSS, and Jet Lag Questionnaire (JLQ) on Day 2, 3, and 4. All outcomes were centrally assessed.

The safety assessments include adverse events (and serious adverse events); laboratory evaluations (hematology, blood chemistry and urinalysis values); vital signs; physical examinations and electrocardiograms.

### Statistical analysis

Sample size was not determined based on any statistical considerations. The sample size of up to 48 participants was chosen based on the observational phase data. Due to the complexity of the study, and slow recruitment rate, the study was terminated before it reached the original enrollment goal of 48 participants, with only 25 participants completing both phases of the study (tasimelteon *n* = 13, placebo *n* = 12).

Efficacy analyses were based on the intent-to-treat (ITT) population, which included any participant randomized into the study that received at least one dose of the study drug and that completed at least one post-baseline efficacy measurement (at least 1 night of PSG data) while on study medication. The primary objective outcome measure analysis used analysis of variance (ANOVA) with treatment and time-zone-shift (5- or 8-h, as a categorical variable) as main effects. As needed, the values collected in the observational phase at corresponding time points were used as baseline. All continuous endpoints were analyzed using ANOVA, Analysis of Covariance (ANCOVA) and Wilcoxon rank-sum test. Categorical variables were analyzed using Barnard's exact test. The difference between treatment groups analyzed using ANOVA method was summarized by the difference between the least squared (LS) means, the associated two-sided 95% confidence interval (95% CI), and the *p*-value for the difference in LS means. For the analysis done by Wilcoxon rank-sum test, the sum and mean of the rank were presented along with *p*-value calculated using exact method. Safety analyses were based on the safety population, which included any participant randomized into the study that receives a dose of study drug. SAS version 9.2 or higher was used in all analyses. A data monitoring committee did not oversee the study. This trial is registered on ClinicalTrials.gov under NCT02560103 (observational phase) and NCT03291041 (evaluation phase).

### Role of funding source

The funder of the study designed the study, performed data analysis, data interpretation, and writing of the report. The funder of the study did not participate in data collection or data monitoring. The corresponding authors had full access to all of the data in the study and had final responsibility for the decision to submit for publication.

## Results

**Figure 3:**
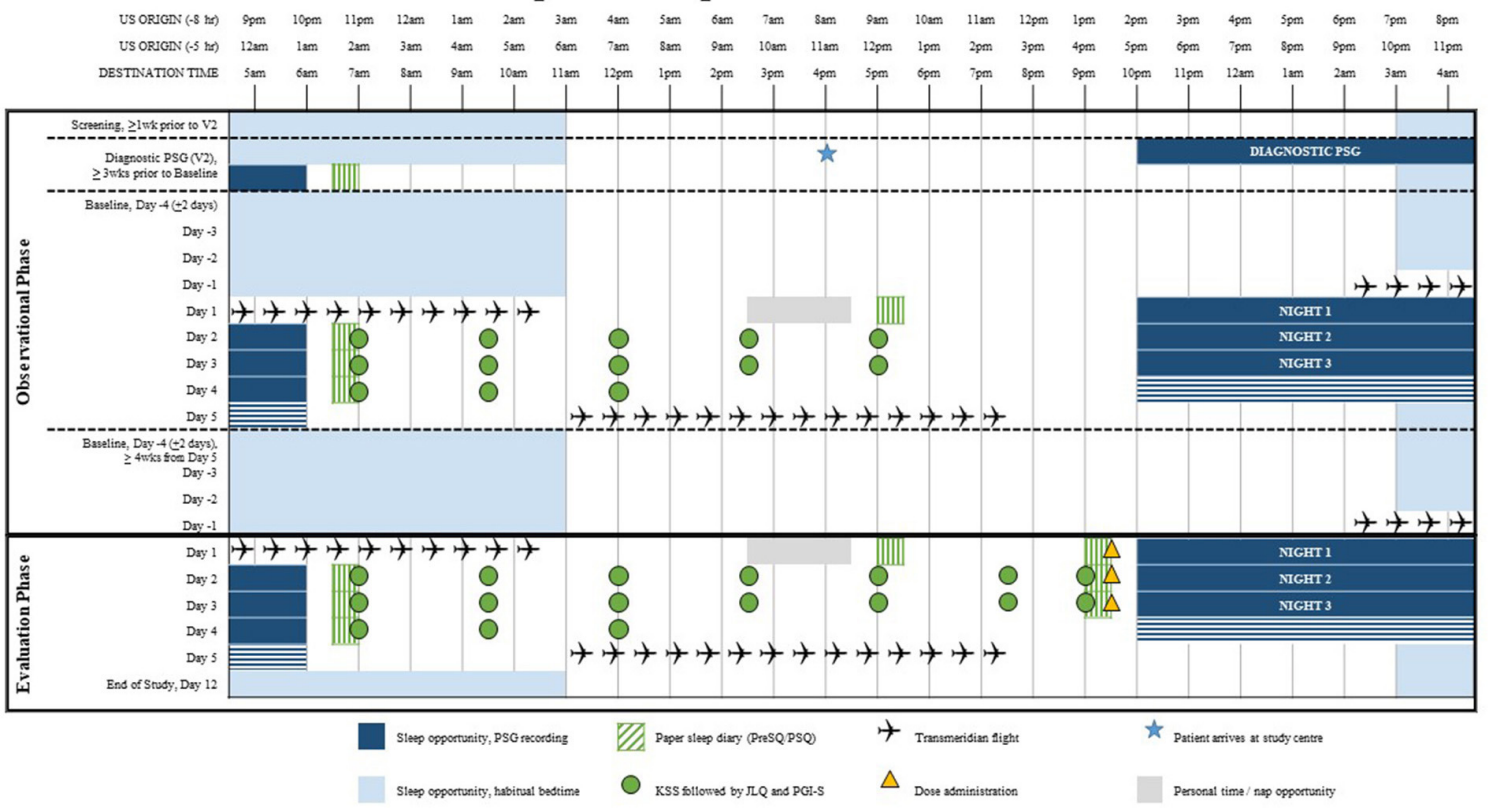
Trial profile.

Between September 24, 2015 and November 6, 2017 a total of 446 participants were assessed for eligibility in the JET study. One hundred and eighteen (26.5%) participants met the inclusion criteria and completed the observational phase. Sixty-eight (15.2%) participants continued to meet the inclusion criteria of which 25 (5.6%) were randomized into the evaluation phase. Following randomization, 25 (100%) participants completed the study and were included in the ITT population analysis (Figure 3).

Baseline demographic characteristics were similar in the two treatment groups. Of the 25 randomized participants, 10 (40%) were male. The average age was 49.8 (SD 14.54), and average BMI was 25.2 (SD 3.15). Baseline values for TST_2/3_ and KSS were similar among the two treatment groups. The racial breakdown was 16 (64.0%) White, 4 (16.0%) Black or African American, 5 (20.0%) Asian, 0 (0.0%) Native Hawaiian or Other Pacific Islander, 0 (0.0%) American Indian or Alaska Native, and 0 (0.0%) Other. The number of participants in the 5- and 8-h phase advance cohorts were similar between treatment groups. Table 1 lists the baseline characteristics of the JET study participants.

**Table 1:** Baseline characteristics for the ITT population.

	**Tasimelteon 20 mg (*N* = 13)**	**Placebo (*N* = 12)**	**Total (*N* = 25)**
**Age in years, mean (SD)**	51·3 (13.47)	48·2 (16.06)	49·8 (14.54)
**Sex**			
Male	4 (30.8%)	6 (50.0%)	10 (40.0%)
Female	9 (69.2%)	6 (50.0%)	15 (60.0%)
**Baseline BMI (kg/m** ^**2**^ **), mean (SD)**	25·0 (3.3)	25·4 (3.1)	25·2 (3.1)
**Ethnicity**			
Hispanic or Latino	1 (7.7%)	0 (0.0%)	1 (4.0%)
Not Hispanic or Latino	12 (92.3%)	12 (100.0%)	24 (96.0%)
**Race**			
White	10 (76.9%)	6 (50.0%)	16 (64.0%)
Black or African American	1 (7.7%)	3 (25.0%)	5 (20.0%)
Asian	2 (15.4%)	3 (25.0%)	5 (20.0%)
Native Hawaiian or Other Pacific Islander	0 (0.0%)	0 (0.0%)	0 (0.0%)
American Indian or Alaska Native	0 (0.0%)	0 (0.0%)	0 (0.0%)
Other	0 (0.0%)	0 (0.0%)	0 (0.0%)
**Phase advance cohort**			
5-h	4 (30.8%)	3 (25.0%)	7 (28.0%)
8-h	9 (69.2%)	9 (75.0%)	18 (72.0%)

Baseline is defined as the last non-missing measurement prior to start of study drug.

Data are n (%), or mean (SD); BMI, Body Mass Index.

**Figure 4:**
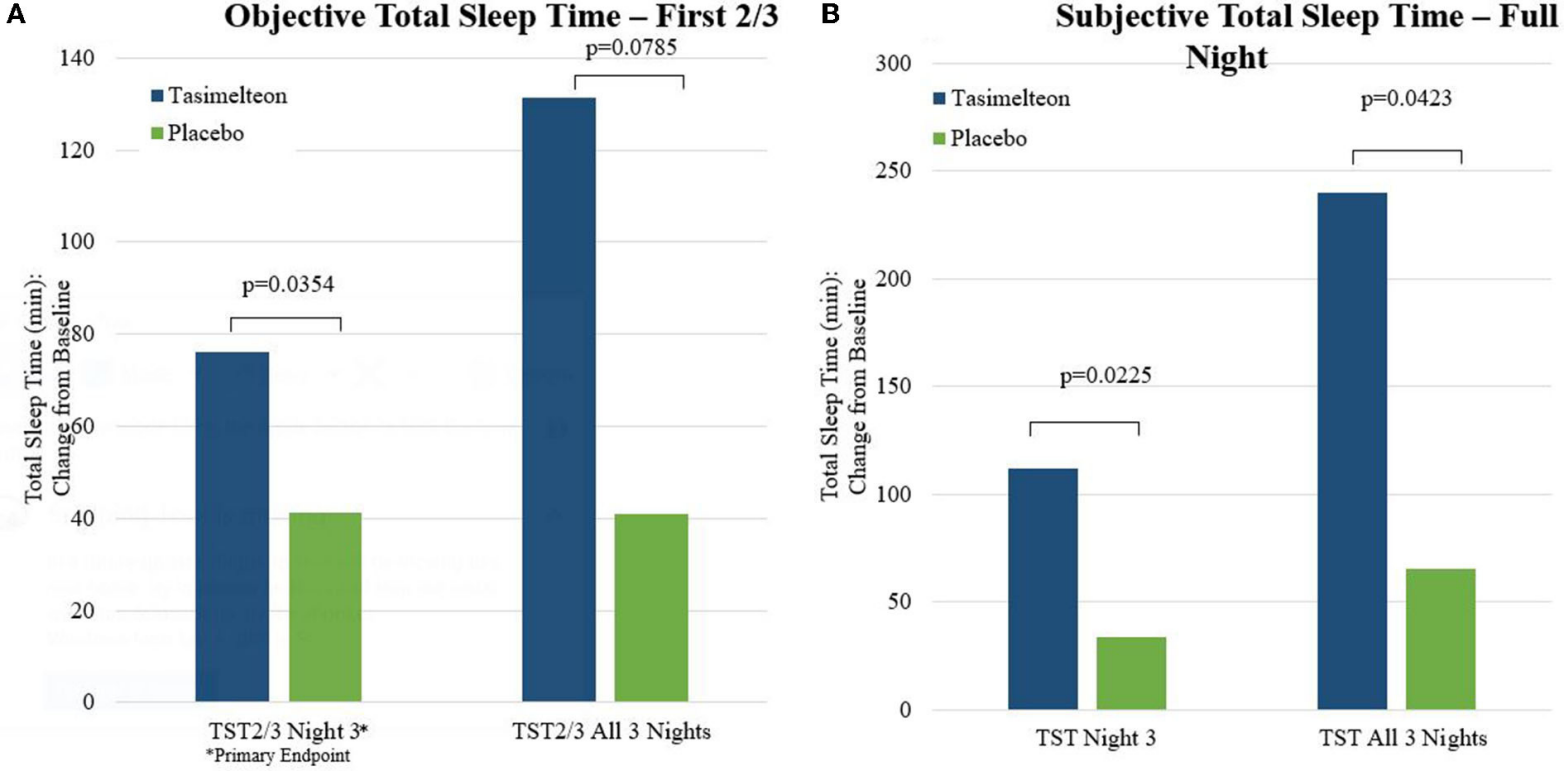
Total sleep time—objective and subjective measures.

Tasimelteon treatment resulted in an increase in objective total sleep time in first two-thirds of the night (TST_2/3_) change from baseline on Night 3 of 34.8 min (95% CI: 2.6–66.9, *p* = 0.0354). TST_2/3_ on Night 3 without adjusting for baseline was also analyzed, with a statistically significant difference favoring tasimelteon of 37.2 min (95% CI: 3.6–70.9, *p* = 0.0315) (Figure 4).

**Figure 5:**
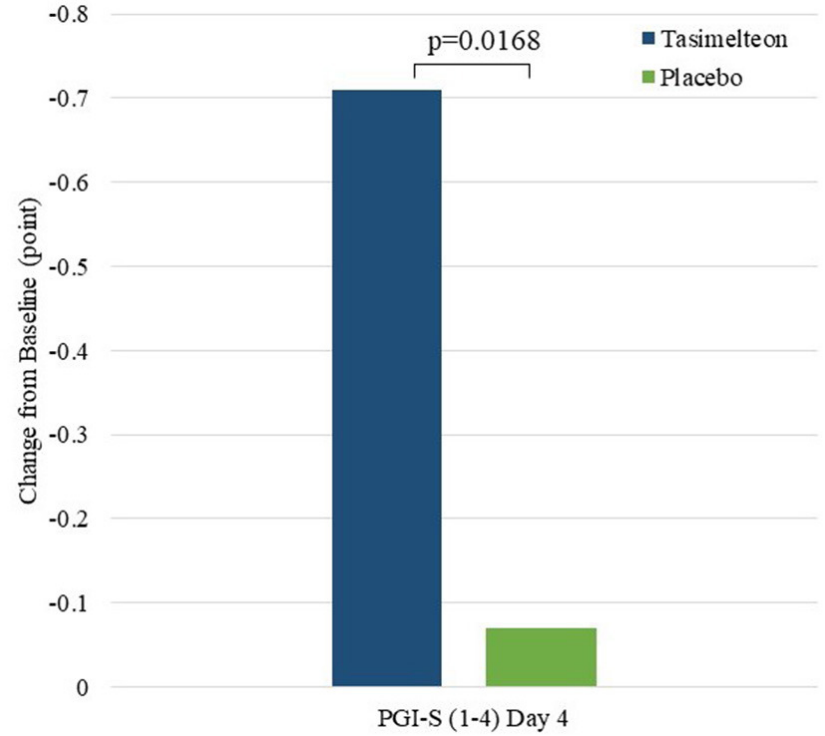
Global functioning (Day 4)—change from baseline.

Patient Global Impression of Severity (PGI-S) on Day 4 was significantly improved for tasimelteon compared to placebo with a difference of −0.63 (95% CI: −1.1 to −0.1, *p* = 0.0168) (Figure 5). Subjective Total Sleep Time (TST_s_) on Night 3 (measured on Day 4) was significantly improved for tasimelteon compared to placebo with a difference between treatment groups of 78.45 min (95% CI: 12.2–144.7, *p* = 0.0225). Subjective Total Sleep Time (TST_s_) on All 3 Nights (measured on All 3 Days) was significantly improved for tasimelteon compared to placebo with a difference between treatment groups of 174.9 min (95% CI: 6.7–343.2, *p* = 0.0423). Subjective Latency to Persistent Sleep (LPS_s_) change from baseline on Night 3 (measured on Day 4) was significantly improved for tasimelteon compared to placebo with a difference of −26.55 min (95% CI: −51.0 to −2.1, *p* = 0.0347). Subjective Sleep Quality (SQ_s_) on Night 3 (measured on Day 4) was significantly improved for tasimelteon compared to placebo with a difference of 0.95 (95% CI: 0.2, 1.7, *p* = 0.0198). All the other secondary outcomes are listed in Tables 2, 3.

**Table 2:** Summary of endpoints for the ITT population.

**Assessment**	**Placebo (*N* = 12)**	**Tasimelteon 20 mg (*N* = 13)**	**Difference (95% CI)**	***P*-value**
**TST**_**2/3**_ ^y^				
Night 1 (min)	234.10 (18.237)	266.64 (17.108)	32.54 (23.638)	0.1825
Night 2 (min)	245.12 (19.454)	250.59 (18.249)	5.47 (25.215)	0.8304
Night 3 (min)^*^	238.65 (12.505)	275.89 (11.731)	37.24 (16.209)	0.0315
**TST**_FN_ ^y^
Night 1 (min)	363.33 (24.042)	387.76 (22.553)	24.43 (31.162)	0.4414
Night 2 (min)	57.59 (26.566)	375.10 (24.921)	17.51 (34.433)	0.6161
Night 3 (min)	365.52 (23.007)	381.50 (21.582)	15.98 (29.820)	0.5974
**TST**_**S**_ ^z^				
Night 1, measured on Day 2 (min)	356.82 (31.919)	434.35 (29.943)	77.53 (41.372)	0.0743
Night 2, measured on Day 3 (min)	373.57 (33.347)	396.16 (31.283)	22.59 (43.223)	0.6065
Night 3, measured on Day 4 (min)	368.72 (28.754)	441.70 (26.974)	72.99 (37.270)	0.0630
**LPS** ^y^				
Night 1 (min)	32.81 (10.499)	24.55 (9.849)	−8.26 (13.609)	0.5499
Night 2 (min)	35.81 (14.532)	31.15 (13.632)	−4.66 (18.836)	0.8069
Night 3 (min)	32.68 (8.051)	15.10 (7.553)	−17.58 (10.436)	0.1062
**PGI-S** ^z^				
Night 1, measured on Day 2	2.38 (0.202)	2.26 (0.189)	−0.12 (0.261)	0.6519
Night 2, measured on Day 3	2.03 (0.242)	1.98 (0.227)	−0.05 (0.314)	0.8873
Night 3, measured on Day 4	1.94 (0.249)	1.61 (0.234)	−0.33 (0.323)	0.3145
**KSS** ^z^				
Night 1, measured on Day 2	5.35 (0.578)	5.39 (0.542)	0.04 (0.749)	0.9548
Night 2, measured on Day 3	4.80 (0.651)	4.90 (0.610)	0.10 (0.843)	0.9053
Night 3, measured on Day 4	4.29 (0.622)	3.73 (0.583)	−0.57 (0.806)	0.4899

TST_2/3_, Total Sleep Time in the first two thirds of the night; TST_FN_, Total Sleep Time; TST_S_, Subjective Total Sleep Time; LPS, Latency to Persistent Sleep; PGI-S, Patient Global Impression Scale; KSS, Karolinska Sleepiness Scale.

* Primary Endpoint.

y Objective measure.

z Subjective measure.

**Table 3:** Summary of endpoints for the ITT population (5 vs. 8 h).

	**5 h**				**8 h**			
**Assessment**	**Placebo**	**Tasimelteon 20 mg**	**Difference**	***P*-value**	**Placebo**	**Tasimelteon 20 mg**	**Difference**	***P*-value**
	**(*N* = 3)**	**(*N* = 4)**	**(95% CI)**		**(*N* = 9)**	**(*N* = 9)**	**(95% CI)**	
**TST** $2_{/}^{3}$ ^y^
Night 1 (min)	229.50 (34.058)	272.50 (29.495)	43.00 (−72.82, 158.82)	0.3837	234.72 (20.196)	263.28 (20.196)	28.56 (−31.99, 89.10)	0.3323
Night 2 (min)	289.33 (26.297)	246.00 (22.773)	−43.33 (−132.76, 46.09)	0.2681	219.50 (22.191)	243.556 (22.191)	24.06 (−42.47, 90.58)	0.4545
Night 3 (min)^*^	253.00 (25.154)	276.88 (21.784)	23.88 (−61.66, 109.41)	0.5052	229.39 (13.438)	271.72 (13.438)	42.33 (2.05, 82.62)	0.0406
**TST**_**FN**_ ^y^
Night 1 (min)	364.17 (41.166)	396.00 (35.651)	31.83 (−108.15, 171.82)	0.5842	359.67 (27.286)	381.28 (27.286)	21.61 (−60.19, 103.41)	0.5832
Night 2 (min)	432.00 (25.724)	374.00 (22.278)	−58.00 (−145.48, 29.48)	0.1490	311.94 (31.032)	358.22 (31.032)	46.28 (−46.76, 139.31)	0.3073
Night 3 (min)	408.00 (33.360)	381.50 (28.890)	−26.50 (−139.94, 86.94)	0.5744	339.22 (26.432)	371.39 (26.432)	32.17 (−47.08, 111.41)	0.4022
**TST**_**S**_ ^z^
Night 1, measured on Day 2 (min)	346.67 (39.821)	451.25 (34.486)	104.58 (−30.83, 240)	0.1039	356.67 (38.051)	423.89 (38.051)	67.22 (−46.85, 181.30)	0.2296
Night 2, measured on Day 3 (min)	378.33 (42.753)	415 (37.025)	36.67 (−108.72, 182.05)	0.5454	363.44 (39.750)	380.67 (39.750)	17.22 (−101.95, 136.39)	0.7633
Night 3, measured on Day 4 (min)	395 (26.794)	443.75 (23.204)	48.75 (−42.36, 139.86)	0.2274	351.67 (35.133)	433.89 (35.133)	82.22 (−23.11, 187.55)	0.1174
**LPS** ^y^
Night 1 (min)	68.67 (36.858)	25.88 (31.920)	−42.79 (−168.13, 82.55)	0.4203	10.11 (3.836)	15 (3.836)	4.89 (−6.61, 16.39)	0.3809
Night 2 (min)	15.83 (37.026)	57.13 (32.065)	41.29 (−84.62, 167.20)	0.4377	38.28 (12.622)	16.11 (12.622)	−22.17 (−60.01, 15.67)	0.2322
Night 3 (min)	53.83 (25.449)	12.13 (22.040)	−41.71 (−128.25, 44.83)	0.2704	20.72 (5.134)	12.33 (5.134)	−8.39 (−23.78, 7)	0.2649
**PGI-S** ^z^
Night 1, measured on Day 2	2.48 (0.477)	2.14 (0.413)	−0.33 (−1.95, 1.29)	0.6187	2.36 (0.201)	2.33 (0.201)	−0.04 (−0.64, 0.57)	0.8960
Night 2, measured on Day 3	2.14 (0.584)	2 (0.505)	−0.14 (−2.12, 1.84)	0.8632	1.95 (0.241)	1.94 (0.241)	−0.01 (−0.73, 0.71)	0.9795
Night 3, measured on Day 4	2.33 (0.308)	1.42 (0.267)	−0.92 (−1.96, 0.13)	0.0741	1.78 (0.289)	1.67 (0.289)	−0.11 (−1, 0.76)	0.7912
**KSS** ^z^
Night 1, measured on Day 2	6.76 (1.006)	5.21 (0.871)	−1.55 (−4.97, −1.55)	0.2974	4.54 (0.621)	5.19 (0.621)	0.65 (−1.21, 2.51)	0.4708
Night 2, measured on Day 3	6.47 (1.359)	4.72 (1.177)	−1.76 (−6.38, 2.86)	0.3729	3.83 (0.653)	4.64 (0.653)	0.81 (−1.15, 2.77)	0.3932
Night 3, measured on Day 4	5.89 (0.901)	3.25 (0.781)	−2.64 (−5.70, 0.43)	0.0779	3.48 (0.679)	3.70 (0.679)	0.22 (−1.81, 2.26)	0.8191

TST_2/3_, Total Sleep Time in the first two thirds of the night; TST_FN_, Total Sleep Time; TST_S_, Subjective Total Sleep Time; LPS, Latency to Persistent Sleep; PGI-S, Patient Global Impression Scale; KSS, Karolinska Sleepiness Scale.

* Primary endpoint.

y Objective measure.

z Subjective measure.

The safety analysis included data from 25 participants (tasimelteon *n* = 13, placebo *n* = 12) who were enrolled, randomized, and received multiple oral doses of the double-blind study medication. The most common treatment emergent adverse event reported in the JET study was headache [4 (30.8%) tasimelteon vs. 4 (33.3%) placebo]. The observed TEAEs for the tasimelteon group in decreasing frequency were headache, vomiting, and ear discomfort. There were no serious adverse events reported in this study. Tasimelteon 20 mg was safe and well-tolerated at the doses studied.

## Discussion

The JET study showed tasimelteon to be more effective than placebo in treating Jet Lag Disorder in travelers who flew from the US to the UK. Tasimelteon significantly improved total sleep time of the first two thirds of the night (TST_2/3_) on Night 3, in both objective and subjective measures of sleep. Tasimelteon-treated participants slept 76 min longer on Night 3 during their second trip as compared to their first. Cumulatively over the 3 travel nights of their second trip, tasimelteon-treated participants added 131 min of sleep (TST_2/3_) as compared to the three travel nights of the first trip. The first two-thirds of the night are maximally overlapped with the previous circadian day, including the circadian wake maintenance zone, when the output of the central circadian pacemaker is most likely to interfere with sleep (12). Further, many individuals habitually sleep <8 h per night and therefore wakefulness in the final third of an 8-h interval may represent the normal morning after a sufficient night of sleep (12).

Similar results favoring tasimelteon were seen in outcomes of the Post Sleep Questionnaire (PSQ), including subjective TST, subjective LPS, subjective WASO and subjective SQ. Measures of global function, including PGI-S and the KSS, also favored tasimelteon. We observed a statistically significant correlation between PSG measures on night 3 and subjective measures such as Sleep Quality (corr.pearson = 0.46; *p*-value < 0.01) and PGI-S (corr.pearson = 0.52; *p*-value < 0.007). The statistically significant correlation was not observed between PSG measures and KSS due to a small sample size. But the correlation between KSS and PGI-S as well as SQ was statistically significant. Importantly the magnitude of the total benefit over placebo achieved by tasimelteon treatment was clinically meaningful.

The results of the JET study support the pivotal JET5 and JET8 Phase III studies, which confirmed significant effects in circadian advances of 5- and 8-h, respectively, and add to the body of evidence of the effectiveness of tasimelteon in treating Jet Lag Disorder (11).

Tasimelteon 20 mg was safe and well-tolerated in this study.

One limitation of this study is the relatively small sample size due to the complexity of the study, primarily the very slow recruitment process as well as the high screen failure rate (94%). This reflects the very strict eligibility criteria, which allowed only participants with severe forms of the disorder. Another limitation is the potential for confounding variables to be introduced by the real-world flight that are unrelated to jet lag disorder such as airline cabin pressure, cramped seating, changes in altitude, and possible dehydration due to low humidity levels in the air. These confounders could be reduced by directly assessing the treatment effect of tasimelteon on what is thought to be the underlying cause of the Essential Features of jet lag—the misalignment of the endogenous circadian timing system with that of the external environment—using a phase advance study design. Phase advance studies of 5- and 8-h have been conducted and reported for HETLIOZ® and the results are supportive of the therapeutic effect of HETLIOZ® in that setting (13, 14).

In addition, it is important to consider that transmeridian travel is not the only way to induce jet lag. The term “social jet lag” is increasingly used to refer to sudden shifts in sleep/wake timing due to scheduled obligations (i.e., teenagers waking up for school on Monday at 6 a.m. after sleeping past noon on the weekends). Limiting the study of jet lag to only real flight studies may unintentionally exclude these alternative forms of jet lag. As societal demands on our schedules are commonplace, it is important to address jet lag as an important global health issue impacting many people for which there is no approved treatment.

Overall, the results of the JET study demonstrate the efficacy of tasimelteon in the treatment of the symptoms of Jet Lag Disorder following a flight. The results of the study strongly suggest that tasimelteon may be an effective therapeutic tool in the treatment of individuals with JLD.

## Data availability statement

The raw data supporting the conclusions of this article will be made available by the authors, without undue reservation.

## Ethics statement

The studies involving human participants were reviewed and approved for VP-VEC-162-0101 (JET observational phase): Copernicus Group IRB Tracking #: MED1-15-458 Yorkshire and The Humber—Sheffield Research Ethics Committee REC Reference #: 15/LO/1732 IRAS Project ID: 189396 For VP-VEC-162-2102 (JET evaluation phase): Copernicus Group IRB Tracking #: MED1-16-286 Yorkshire and The Humber—Sheffield Research Ethics Committee REC Reference #: 16/YH/0282 IRAS Project ID: 209224 EudraCT Number: 2016-002213-21. The patients/participants provided their written informed consent to participate in this study.

## Author contributions

CP, GB, CX, and MP contributed to the study concept and design. CX, CP, and MP developed the statistical analysis plan. CP wrote the report in collaboration with SS, EC, and MF. CP revised the report with participation from all authors. All authors reviewed and approved the report before submission.

## Funding

This study was funded by Vanda Pharmaceuticals Inc.

## Conflict of interest

Authors CP, VP, EC, MF, SS, CX, GB, and MP are employees of Vanda Pharmaceuticals Inc. This study received funding from Vanda Pharmaceuticals Inc. The funder had the following involvement with the study: The funder of the study designed the study, performed data analysis, data interpretation, and writing of the report. The funder of the study did not participate in data collection or data monitoring. Author SS had full access to all of the data in the study and had final responsibility for the decision to submit for publication.

## Publisher's note

All claims expressed in this article are solely those of the authors and do not necessarily represent those of their affiliated organizations, or those of the publisher, the editors and the reviewers. Any product that may be evaluated in this article, or claim that may be made by its manufacturer, is not guaranteed or endorsed by the publisher.
